# Treatment of Dry Eye Disease (DED) in Asia: Strategies for Short Tear Film Breakup Time-Type DED

**DOI:** 10.3390/pharmaceutics15112591

**Published:** 2023-11-05

**Authors:** Atsushi Kawahara

**Affiliations:** Yoshida Eye Hospital, 2-31-8, Hondori, Hakodate 041-0851, Hokkaido, Japan; atsusi-k@coral.plala.or.jp; Tel.: +81-138-53-8311

**Keywords:** dry eye disease (DED), dry eye, dry eye syndrome, tear film, diquafosol, cyclosporine, supplement, contact lens, serum, meibomian gland dysfunction

## Abstract

Dry eye disease (DED) is a multifactorial disorder in which tear fluid homeostasis is lost, resulting in increased tear film osmolarity and ocular surface irritation. In Asia, the short tear film breakup time-type DED, which has become a global problem in recent years, is common. While the mainstay of DED treatment in the West is the suppression of inflammation, the first goal of treatment is the stabilization of the tear film in Asia. To date, artificial tears and steroid eye drops have been the main treatment for DED. However, artificial tears require frequent administration of eye drops and thus pose adherence problems, while steroids have problems with side-effects (cataracts, increased intraocular pressure). This review evaluates the new generation therapies in Asia based on what is known about them and demonstrates that they are more effective for DED than traditional therapies such as artificial tears and steroids. Based on considerations, it is proposed that the optimal treatment for the short tear film breakup time-type DED is the initial application of mucin-secretion-enhancing eye drops (long-acting diquafosol) and oral supplements; and if additional treatment is needed, cyclosporine eye drops and the adjunctive therapies presented in this review are added.

## 1. Introduction

Dry eye disease (DED) is a multifactorial disorder in which tear fluid homeostasis is lost, i.e., when the chemical composition and function of the tear fluid are out of balance [1], and is characterized by increased osmotic pressure in the tear film and ocular surface inflammation [2,3]. The prevalence of DED is more than 30% in Asia [4] and approximately 14% in the USA [5], is more common in women and tends to increase with age [6]. Increased tear osmolarity and ocular surface inflammation are characterized by increased tear evaporation and decreased production. The release of inflammatory mediators via T lymphocytes as a physiological defense mechanism against these and the resulting vicious cycle of inflammation on the ocular surface are considered in the West to be the main pathogenesis of DED. Therefore, the main treatment in the West is anti-inflammatory treatment. On the other hand, the short tear film breakup time-type DED, in which inflammation is not the main pathology, has attracted more attention in recent years. This type of DED is most common in Asia.

**Figure 1:**
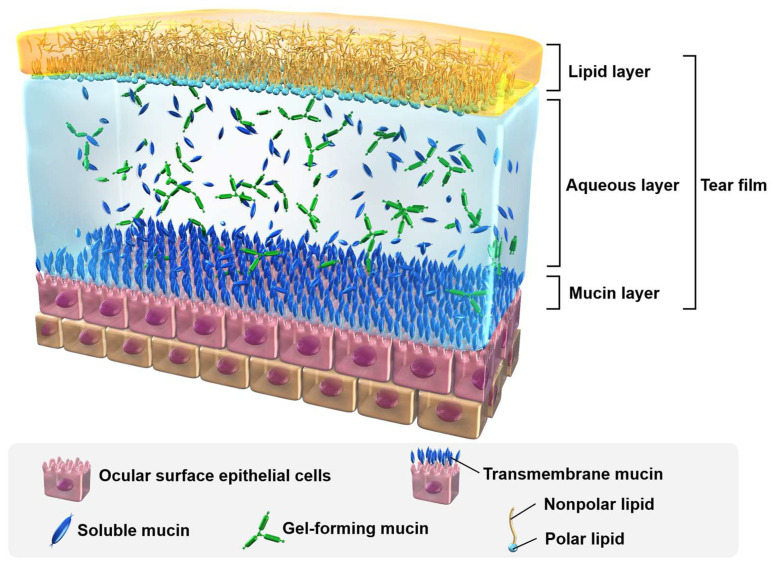
The structure of tear film.

The ocular surface is the mucous membrane most exposed to external stimuli in humans. The tear film covers the mucosa and acts as a barrier to protect it from oxidative stress and free radicals [7]. The structure of the tear film is shown in Figure 1. The outer layer of the tear film is the lipid layer, which is formed by secretions from the meibomian glands present in the eyelid. The lipid layer consists of two layers, polar and nonpolar lipids. The nonpolar lipid layer is in contact with the external environment as the outer lipid layer, while the polar lipid layer is in contact with the water layer as the inner layer, stabilizing the entire lipid layer [8]. The nonpolar lipid layer forms an optically suitable ocular surface and a barrier against the stresses of the external environment and plays a particularly important role in tear film stability as the component that controls tear evaporation [9]. Beneath the lipid layer is an aqueous layer containing water, electrolytes, metabolites, and proteins, which smooths the ocular surface by maintaining an appropriate osmotic pressure. The mucin layer lies beneath the aqueous layer, and the mucins in the tear fluid are composed of soluble, gel-forming, and transmembrane mucins [10]. Soluble and gel-forming mucins are present in the aqueous layer and give the tear fluid its pseudoplastic properties. Mucins in the aqueous layer also act as surfactants to diffuse lipids over the aqueous layer, preventing excessive evaporation of the tear fluid. Transmembrane mucins are present on the surface of epithelial cells and express sugar chains that link the tear film to the ocular surface epithelial cells and stabilize the tear film. Thus, when one (or more) of the tear film is weakened and exposed to an environment in which the tear fluid evaporates, the mechanisms that eliminate oxidative stress and other factors are weakened. The short tear film breakup time-type DED is a DED in which this tear fluid breakdown is the primary pathogenesis. The Asian Dry Eye Society classifies DED based on etiology into three categories: evaporative, aqueous-deficient, and decreased wettability categories. Evaporative and decreased wettability DEDs are the short tear film breakup time-type DED caused by mucin dysfunction, deficiency, or meibomian gland dysfunction, and are the main types of DEDs in Asia [11]. The most well-known method of assessing the severity of DED is the classification by the International Dry Eye Work Shop, which determines levels 1 to 4 based on factors such as subjective symptoms, keratoconjunctival damage, Schirmer test values, tear breakup time and eyelid (meibomian gland) status, which can be used for clinical management [2].

In DED, therapy is essentially started with ophthalmic solutions. In the past, artificial tears have been used, but with the short residence time of artificial tears on the ocular surface, the improvement in both subjective symptoms and objective findings were temporary [12]. As an anti-inflammatory agent, corticosteroid eye drops have been shown to have therapeutic efficacy, but problems with side-effects (cataracts, increased intraocular pressure) limit their application in long-term treatment [13]. In addition, recent randomized controlled trials [14,15] have shown that nonsteroidal anti-inflammatory eye drops are less effective for DED, as well as the side-effect of reduced corneal perception. Thus, a drug that is more effective than artificial tears and does not have the severe side-effects observed with corticosteroids and nonsteroidal anti-inflammatory drugs were long awaited, and sodium hyaluronate ophthalmic solution was developed. Sodium hyaluronate bound to fibronectin in tear fluid accelerates the adhesion and extension of corneal conjunctival epithelial cells, thereby ameliorating epithelial damage [16], and its conjugates retain water, thereby resulting in greater water retention [16]. However, the stabilizing effect of the all-important tear fluid layer was weak, and the therapeutic effect of DED was often not observed [17].

The most common type of DED in Asia is the short tear film breakup time-type DED, which is characterized by an unstable tear film [18]. In 2007, the International Dry Eye Work Shop defined DED [2] as “a multifactorial disorder of the tear fluid and ocular surface causing ocular distress, impaired visual function, and an unstable tear film that can injure the ocular surface. Dry eye is associated with hyperosmolarity in the tear film and inflammation on the ocular surface.” In 2014, the Asia Dry Eye Society defined the criteria for the diagnosis of DED [19] as “a multifactorial disorder characterized by instability of the tear film, which can cause a variety of symptoms and/or visual impairment and may be associated with ocular surface damage.” In particular, this diagnostic criterion suggests that the target of DED treatment is stabilization of the tear film. In recent years, new eye drops and new treatments for DED have been developed, and their clinical results have been reported from Asia. The aim of this review is to assess the latest treatment for DED in Asia and to suggest the optimal treatment for the short tear film breakup time-type DED, which has become a global problem in recent years.

## 2. Literature Search

PubMed was used to systematically search the literature simultaneously for randomized controlled trials, nonrandomized trials and review articles on therapies for DED. I read the titles and abstracts of the articles retrieved, extracted the full text of the original articles or review articles written in English, identified new findings from these articles to determine, particularly with regard to eye drops, whether they are approved in Asia for the treatment of DED, and determined the treatments reviewed in the present paper. Articles published only as abstracts or conference posters or not published in English were excluded. From the excluded papers, treatments that have attracted attention in recent years were extracted and presented in the Expected future therapy section.

## 3. Major Therapy (New Generation Eye Drops)

Artificial tears have played a central role in DED treatment to date [20]. Artificial tears are a tear fluid replacement that allows DED patients to supplement normal human tear fluid. Further, most artificial tears are not prescribed medicines, but are categorized as medical equipment or medicines that can be bought on the market over the counter. Because of these advantages, it was widely adopted and is continuing to be used by many patients. Although a large range of artificial tears have been researched and advanced [16,20], their effectiveness is, regrettably, transient. Moreover, the physical properties of eye drops, which affect comfort of use and blurred vision, are becoming increasingly important, but unlike prescription drugs, the physical properties of artificial tears (the content of viscosity-enhancing agents, electrolytes, osmo-protectants, oily agents, antioxidants and preservatives, etc., and their mechanical and pharmacological effects) are not always available from the manufacturer [21]. This may make it difficult to select an artificial tears solution that is appropriate for the medical condition. For these reasons, treatment relying on artificial tears has limitations, and the new generation of DED treatment eyedrops is in the spotlight. Rebamipide, approved in Japan, is a mucin secretagogue eye drop and a new-generation DED treatment [22], but it was not included in this section because there were no reports containing findings of superior efficacy compared to diquafosol. The treatments in this section are listed in Table 1.

### 3.1. Diquafosol

Diquafosol (a dinucleotide derivative), which acts on P2Y_2_ receptors, is a mucin secretagogue eye drop [23]. This receptor is represented in the corneal, conjunctival, lacrimal gland secretory, pararectal lacrimal gland secretory, and meibomian gland secretory epithelium [24]. This receptor stimulation enhances mucin and water secretion via Ca^2+^ and Cl^−^ channels [25,26] and also increases the thickness of the lipid layer within the tear film [27,28]. Tear fluid production becomes increased for 5 to 30 min after diquafosol eyedrops [26], but this is unrelated to the performance of the lachrymal gland [29]. On the other hand, the tear fluid augmentation period of artificial tears is 5 min [26], which also confirms the tear fluid augmentation effect of diquafosol. Further, diquafosol augments the lipid layer within the tear film for a maximum of 60 min [30]. Diquafosol enhances mucin, water, and lipids to maintain a stable tear film. In addition to that, diquafosol stimulates epithelial cell proliferation and repair by promoting the phosphorylation of epidermal growth factor receptors and extracellular signal-regulated kinases [31]. Indeed, it has been confirmed that diquafosol reverses apoptosis of corneal epithelial cells [32]. A multicenter clinical trial reported that diquafosol significantly decreased corneal epithelial disorders by increasing tear secretion and corneal epithelial barrier function [33]. This trial showed that it was effective for tear fluid deficiency-type DED as well as short tear film breakup time-type DED [33]. Diquafosol ophthalmic solution has also been reported to improve practical vision as well as subjective symptoms of DED (dryness, foreign body sensation, eye pain, photophobia and blurred vision) [34]. Diquafosol’s most frequent adverse reaction is eye irritation, with up to 12.5% reported [35], but most are mild and there are no other adverse events observed that are problematic. Thus, diquafosol ophthalmic solution represents a useful new generation eyedrop for the treatment of DED, but the frequency of the drops, which is six times a day, hinders the continuity of treatment [36]. For this reason, in order to achieve good adherence, the long-acting diquafosol was long awaited and was approved in Japan in 2022. This new type of diquafosol was made possible by the addition of polyvinylpyrrolidone to conventional diquafosol to extend its action [37]. The number of recommended eye drops is 3 times a day, and has been demonstrated to be comparable to the efficacy and safety of conventional diquafosol [37].

### 3.2. Cyclosporine

Inflammation is both a cause for DED and a condition induced due to DED [19]. Animal studies have confirmed that dry stress induces the release of T helper 1 type cytokines on the ocular surface resulting in the disruption of the corneal epithelial barrier associated with T helper 17 cells [38]. Pro-inflammatory cytokines are increased in the tear fluid in patients with DED [39] and the inflammation reduces the production and secretion of mucin in the corneal conjunctival epithelium [40] and destabilizes the tear film. Consequently, inflammation must be controlled to ameliorate the destabilized tear film. Cyclosporine, an immunosuppressant drug to regulate inflammation, is well known as an efficient therapeutic agent for DED [41]. Normal tear fluid contains transforming growth factor-β and other anti-inflammatory factors secreted by the lacrimal gland and conjunctival goblet cells, and cyclosporine increases the amount of transforming growth factor-β [42]. In addition, cyclosporine reduces the infiltration of lymphocytes in the lacrimal gland and conjunctival organization and suppresses the development of inflammatory mediators [43]. For patients suffering from mild DED, therapy by cyclosporine has been demonstrated to decrease inflammation-induced organization injury [44]. Especially significant is the fact that Ikervis^®^ can be achieved in only one eye drop per day. Moreover, no evidence has been reported to indicate systematic resorption of cyclosporine following ophthalmic administration [45]. Based on the foregoing, the new generation of cyclosporine ophthalmic solutions is a viable choice to treat DED. Inflammation within the short tear film breakup time-type DED, which predominates in Asia, however, appears to be secondary to the pathogenesis of the disease, and therefore is not thought to be a core component of DED therapy in Asia [19]. Thus, cyclosporine has been indicated as an efficient therapeutic modality rather than alone, when combined with a mucin secretagogue [46]. Also, as heat and stinging pain during eyedrops are observed side-effects of cyclosporine, although minor, this is considered to be a challenge for the future.

## 4. Adjunctive Therapy

Currently, punctal occlusion is the main adjunctive therapy for DED. The purpose of the therapy is to block the patient’s tear ducts and cause tear fluid to pool over the ocular surface. Because this therapy results in better tear film stabilization [47], tear plugs are a valuable adjunctive therapy, but the adverse effects of plug-induced irritation and dacryorrhea are inevitable [48]. So, new adjunctive therapies are also receiving interest. The treatments in this section are listed in Table 2.

### 4.1. Supplement

Evidence has been reported showing the therapeutic efficacy of supplements for DED [49,50]. In particular, the efficacy of nutritional supplementation with antioxidants and phytochemicals found in maquiberry extract, bilberry extract, and astaxanthin, as well as oral hyaluronic acid and omega-3 fatty acids, for DED has been demonstrated. Maquiberry (Aristotelia chilensis) is rich in anthocyanins with abundant delphinium 3,5-O-diglucoside as a major component, and has strong antioxidant properties. It exerts its therapeutic effect on DED by inhibiting the production of reactive oxygen species in the lacrimal gland and increasing tear secretion by preventing lacrimal gland tissue dysfunction [51]. Bilberry (*Vaccinium myrtillus* L.) is a potent antioxidant due to its high anthocyanin content. Its consumption has been shown to increase lacrimal fluid secretion and improve antioxidant capacity [52]. In addition to this, it has been noted that it may contribute to the improvement of eye strain by relaxing the contraction of the ciliary muscle [53]. Astaxanthin is a similarly potent antioxidant with a special molecular structure. There are polar regions at both ends of the molecule’s ionone ring, which neutralize free radicals to prevent chain reactions [54]. Therefore, astaxanthin has anti-inflammatory, anti-apoptotic, and immunomodulatory effects in addition to its antioxidant effect [55]. Astaxanthin intake improved tear film breakup time, blink frequency, and ocular surface disease index [56]. Furthermore, it has been shown to have preventive and therapeutic effects against eye strain, age-related macular degeneration, diabetic retinopathy, and glaucoma [57]. Oral treatment with high-molecular-weight hyaluronic acid has already been introduced to reduce joint pain in osteoarthritis of the knee and to improve moisture retention in dry skin, and it has moisturizing and anti-inflammatory properties [17]. Hyaluronic acid is a macromolecular polysaccharide formed by repeating polymeric disaccharides (D-gluconic acid and N-acetyl-D-glucosamine). When hyaluronic acid is ingested orally, it is absorbed from the intestine, incorporated into the lymphatic fluid, and transferred to tissues without degradation. After translocation into tissues, it suppresses T helper 1-associated inflammation by enhancing interleukin-10 production, anti-inflammatory cytokines, upregulating suppressor of cytokine signaling 3 expression, and downregulating pleiotrophin expression suppresses T helper 1-associated inflammation [17]. Omega-3 fatty acids include n-3-eicosapentaenoic acid, docosahexaenoic acid, and alpha-linolenic acid, which have reportedly been effective in DED treatment due to their anti-inflammatory and neuroprotective effects [58]. On the other hand, the DREAM trial [59] found no significant difference in treatment effect between the omega-3 fatty acid and placebo groups, but reported that omega-3 fatty acid intake improved the symptoms and signs of DED. Thus, supplement therapy is considered effective for DED, but increasing intraocular bioavailability may be a challenge.

### 4.2. Therapeutic Contact Lens

Therapeutic contact lenses are widely utilized for corneal and ocular surface diseases [60]. Soft lenses and scleral lenses are used and have two roles: bandage contact lenses to promote corneal healing and ophthalmic drug delivery systems. Bandage lenses serve as a scaffold for corneal healing and promote epithelialization by enhancing the distribution of tear fluids to the ocular surface. It has been shown to be effective in the treatment of neurotrophic keratitis, ocular chemical injury, and graft-versus-host disease [60]. Bandage lenses are also useful not only after corneal surgery but also after cataract surgery [61,62] and glaucoma surgery [63]. Therapeutic contact lenses for the treatment of DED have been particularly touted an alternate ophthalmic medication delivery system which increases medication residence time in the ocular, increases bioavailability, and allows for more convenient and effective treatment [64]. Contact lens is separated from the cornea with a thin layer of liquid known as the posterior tear fluid layer of the lens. The clearing duration of the posterior tear fluid layer of soft contact lenses is approximately 30 min [65]. In this way, the ophthalmic medication ejected from contact lenses has a retention time on the anterior surface of the cornea of a minimum of 30 min, in comparison to 2 min for ophthalmic solutions. With increased retention time, the bioavailability of the drug could possibly rise up to 50%, in comparison to 1–5% for ophthalmic solutions [66]. This may improve therapeutic efficacy for DED, control drug fluctuations, reduce dosage, and reduce side-effects [67]. Thus, therapeutic contact lenses are a useful adjunctive therapy in the treatment of DED, but the challenge is the potential for ocular surface infections that can occur due to constant contact lens wear. Prolonged wear for treatment increases the likelihood of developing microbial keratitis, which can worsen if the patient does not practice correct contact lens management [68].

### 4.3. Human Umbilical Cord Serum Eye Drops

Autologous serum ophthalmic solutions reportedly are efficacious against severe DED, reoccurring corneal erosions, and graft-versus-host disease [69]. Growth factors such as epidermal growth factor, acid and basic fibroblast growth factor, platelet-derived growth factor, hepatocyte growth factor, vitamin A, transforming growth factor, substance P, insulin growth factor, nerve growth factor, fibronectin and serum anti-protein enzymes have been found in autologous serum [70]. By promoting proliferation, differentiation and maturation of the surface epithelium, such growth factors ameliorate corneal epithelial disorders. They also cause anti-inflammatory effects. Human umbilical cord serum has a higher level of growth factors than autologous serum, which may provide a more powerful treatment effect and make it suitable for Stevens–Johnson syndrome and ocular chemical injury, in addition to the previously mentioned disorders that are effective with autologous serum eyedrops [70,71]. Nevertheless, since some reports [72] have shown that autologous serum eye drops did not improve corneal epithelial injury much, its ability to ameliorate epithelial injury may be questionable. In light of the above, it can be concluded that human umbilical cord serum is a safe and effective adjunct in severe DED, mainly due to its potent anti-inflammatory action. Issues with this therapy are that the collection of cord blood is so complex that it can only be produced in facilities equipped with obstetric services and microbiological assessment with strict preservation protocols; the risk of allergy and the possibility of parenteral infection must always be considered; and there are legal and ethical concerns with cord blood therapy. The benefits of treatment may, however, outweigh the limitations, depending on the patient’s medical condition, and routine use can be recommended.

### 4.4. Intense Pulsed Light

Intense pulsed light treatment was originally performed for the cosmetic community as well as for dermatological diseases (hypertrichosis, benign cavernous hemangiomas, venous malformations, telangiectasias, pigmented lesions, and others) [73]. Intense pulsed light instruments deliver heat selectively to certain structural targets using xenon flashlamps releasing intense polychromatic light with wavelength, depth of penetration, and target areas tuned from the visible to the infrared spectrum [74]. The improvement in DED when patients with facial rosacea underwent intense pulsed light treatment suggested that it could be applied to the treatment of meibomian gland dysfunction [75], and subsequent studies confirmed that intense pulsed light treatment is an effective treatment for DED associated with meibomian gland dysfunction [76,77,78,79,80]. The light intensity of the intense pulsed light energy is transformed into thermal energy when taken up by the chromophore, which solidifies surface vessels, resolving eyelid margin telangiectasias and inhibiting ocular surface inflammation [81]. It has been shown that these responses reduce inflammatory markers in the tear fluid of patients with meibomian gland dysfunction-related DED [76]. In these respects, it appears that intense pulsed light treatment is a valuable adjunctive treatment for DED related to meibomian gland dysfunction. Yet, there is little variation in the energy and pulse intensity of conventional intense pulsed light treatment devices, making it difficult to tailor the treatment to the severity of the meibomian gland dysfunction. Additionally, due to the thermal effects of intense pulsed light treatment, bruising may occur on the skin around the eyes of the individual patient. Improvements in the new generation of intense pulsed light devices have eliminated these disadvantages and improved efficacy and safety [82].

### 4.5. Lid Debris Debridement

Lid debris debridement is the removal of scabs from bacterial biofilm and eyelashes that have accumulated at the eyelid margin and processed with the BlephEx system (RySurg, Fort Worth, FL, USA) [83]. The therapy has been pioneered from the notion that bacterial biofilms cause meibomian gland dysfunction [84]. The biofilm is produced by *Staphylococcus epidermidis and aureus*, which form the bacterial flora of the conjunctival sac and lid margin, and accumulate through the years [85]. If the amount is over a certain level, it activates quorum sensing genes [86], which produce toxins and virulence factors that assault host organizations, leading to a vicious cycle of further expansion of the biofilm [87]. Given the notion that such inflammation causes meibomian gland dysfunction and meibomian gland dysfunction-related DED, the therapy is the therapeutic option of choice for preventing the development and exacerbation of these conditions. In addition, it has recently been reported that this Lid debris debridement in combination with meibomian gland expression additionally ameliorates meibomian gland dysfunction-related DED [88]. The mechanism is that the combined use of meibomian gland expression significantly lifts the obstruction of the meibomian gland ducts, reducing the lipase levels generated by bacteria in the eyelids, improving meibum properties and normalizing the tear lipid layer. As such, lid debridement can be a valuable adjunctive therapy; however, the challenge is that it currently requires regular treatment and there is a lack of clarity on the number of sessions and their duration. Also, side-effects include eyelid margin irritation/erythema and abrasions.

### 4.6. Vectored Thermal Pulsation System

Vectored thermal pulsation system (Lipiflow^®^) is a novel approach that combines thermal therapy and massage for meibomian gland dysfunction. This treatment is useful in the treatment of obstructive myobome gland dysfunction, relieving the obstruction of the meibomian glands and increasing the flow of the secretory product, mibum, thus stabilizing the tear film. The lipid secretions of normal meibomian glands are a clear oily liquid with a melting point of 32 °C [89]. However, when the composition of lipid secretions in dysfunctional meibomian glands changes, they turn into a cloudy liquid or an opaque, toothpaste-like substance [90], and the melting point increases to 35 °C [89]. The LipiFlow^®^ is applied in-hospital with a doctor or technician who applies the procedure over a 12-min period by applying regulated heat to the internal lid surface and intermittently applying pressure to the external lid, causing the cystic glands to release mybam [91]. Side-effects of skin pain and swelling may be observed after treatment. A recent meta-analysis has shown that Lipiflow^®^ treatment improves subjective and objective outcomes of myibomian gland dysfunction and myibomian gland dysfunction-related DED and does not lead to an excess of adverse events. Yet, the lack of a significant difference in efficacy when compared to eyelid hygiene using a combination of warm compress and eyelid massage is a future challenge for this treatment [92].

### 4.7. Intraductal Meibomian Gland Probing

Intraductal meibomian gland probing treats meibomian gland dysfunction by using a small probe to open blocked meibomian glands and promote meibomian secretion. It was developed on the basis of the hypothesis that the symptoms of meibomian gland dysfunction-related DED are due to periglandular fibrosis and tightening of the gland ducts, and that physically widening the gland orifices and gland ducts would provide better symptomatic relief than conventional treatment alone [93]. A sterile probe is inserted under local anesthesia into the duct through the meibomian gland orifice to dilate the duct, reduce intraglandular pressure, and promote normal gland excretion. On the other hand, bleeding during the procedure is recognized as a side-effect. According to a recent review [94], this treatment is effective when combined with other treatments such as intense pulsed light, topical corticosteroids, or conventional therapy [95,96,97], but has not been found to be superior to other treatments when used alone. It also concludes that intraductal meibomian gland probing itself has not yet demonstrated itself to be an efficacious treatment for meibomian gland dysfunction, although it may be an effective choice for patients who do not response to other therapies because it has a mechanism of action that is different from other therapies.

## 5. Expected Future Therapy

This section presents eye drops and adjunctive therapies that are likely for approval and widespread use for the treatment of DED in Asia. Laser acupuncture is an adjunctive therapy that has already been introduced, mainly in China, but is included in this section because clear treatment protocols have not been adequately reported. Future reports from Asia on the results of randomized controlled trials of these therapies in this section are also expected. The treatments in this section are listed in Table 3.

### 5.1. Tacrolimus Ophthalmic Solution

Tacrolimus is a nonsteroidal immunomodulator and has an outstanding safety profile [98]. It has shown excellent anti-inflammatory activity against immune disorders and has been of particular interest for use in ophthalmology. In recent years, it has emerged as a replacement for cyclosporine in the therapy of immunologically mediated inflammatory ocular diseases [99]. On the other hand, tacrolimus ophthalmic solution has shown good results in the treatment of DED [100]. In a recent report [101], the efficacy of cyclosporine and tacrolimus ophthalmic solutions in the therapy of DED was reported to be comparable. However, tingling and burning sensations during eye drops is a recognized side-effect of tacrolimus and is a future issue.

### 5.2. Tanfanercept Ophthalmic Solution

Tanfanercept is an antibody–drug eye drop of a molecule-engineered tumor necrosis factor receptor 1 fragment that targets the suppression of inflammation in DED [102]. Tumor necrosis factor is a key cytokine that mediates the proinflammatory components of a variety of disorders, including DED [103]. Tanfanercept ophthalmic solution was already demonstrated to produce meaningful improvements in DED animal models [104]. In a recent Chinese phase II, single-center, double-blind, randomized, placebo-controlled trial in adult subjects with moderate to severe DED, tanfanercept was safe and tolerable and showed efficacy improvement in DED symptoms compared to placebo [102]. However, the challenge is whether antibody drugs can truly increase intraocular bioavailability, as their large molecular size limits their ability to penetrate ocular tissues.

### 5.3. Lifitegrast Ophthalmic Solution

Integrins facilitate cell–cell interactions and are membrane-spanning receptors; binding of lymphocyte function-related antigen-1 integrin, which is also referred to as CD11a/CD18 or αLβ,2 to cell–cell adhesion molecule-1, activates helper T cells, triggering an inflamed cascade. Integrin antagonists inhibit T cell mobilization and activation and suppress inflammatory responses [105]. Lifitegrast ophthalmic solution, a low-molecular-weight lymphocyte function-related antigen-1 antagonist, has been approved by the U.S. Food and Drug Administration in 2016. Phase 2 [106] and phase 3 trials [107] showed positive outcomes in the therapy of DED, and a systematic review and meta-analysis including these studies also showed a high evaluation in efficacy and safety [105]. On the other hand, lifitegrast is a side-effect of greatest concern and requires caution, as its ophthalmic application causes a distinctive metallic or salty taste, making taste disturbance a side-effect of greatest concern.

### 5.4. Perfluorohexyloctane Ophthalmic Solution

Perfluorohexyloctane ophthalmic solution is a recently introduced product in Europe for the treatment of DED. Perfluorohexyloctane is a semi-fluoroalkane fluid that has initially been employed as a prolonged vitreous replacement in the ophthalmologic field. The chemical compound appears to be inert physically, chemically, and physiologically, and is marginally amphiphilic, colorless, laser stabilized, has a higher density than water, and has extremely poor superficial and interfacial tensions [108]. In addition, because it is a nonaqueous fluid, microorganism propagation is impossible and preservatives are not required [109]. Perfluorohexyloctane ophthalmic solution reportedly reduces symptoms related to dry eye when administered to patients with DED [109]. Perfluorohexyloctane is thought to create an occlusion layer and reduce eyelid shear force upon blinking, thereby avoiding the increase in vaporization resulting in DED, as it increased tear film breakup time and lipid layer thickening in patients with DED [109]. It has also been shown to provide a long-lasting cooling effect on the ocular surface [110]. Yet, the mechanism of action of these eye drops is unknown, and hypersensitivity has been reported as an adverse event, albeit a small one, to this ophthalmic solution [109].

### 5.5. Lactoferrin Ophthalmic Solution

Lactoferrin is a natural occurrence of Fe-binding glycoprotein, produced and secreted on various mammals such as human by mucous membrane epithelial cells and neutrophils, and contained in saliva, milk, tears, and other fluids. With its anti-inflammatory, antioxidant, and antimicrobial actions, lactoferrin plays an essential part in maintaining the ocular surface system’s well-being [111]. Indeed, a significant association of low lactoferrin concentrations in tears with the onset of DED was reported [112]. Recently, a review focusing on the treatment of DED with lactoferrin ophthalmic solution also suggested the possibility of using lactoferrin ophthalmic solution for the treatment of DED [111]. Water-soluble lactoferrin, however, is unstable and is often expelled through the nasolacrimal duct, which inevitably leads to low intraocular bioavailability. Therefore, the technology of lactoferrin-loaded nanoparticles has attracted much attention.

### 5.6. Amniotic Membrane Extract Ophthalmic Solution

For the treatment of ocular surface diseases, amniotic membrane has been used in the form of cryopreservation, lyophilization, and bandage contact lenses [113]. Amniotic membrane derivatives have been tailored to increase their usefulness for clinical use, and methods such as homogenates, frozen or lyophilized extracts, sera and liquids have been used for this purpose [114]. In addition, amniotic membrane extract ophthalmic solution has recently been developed and are beginning to be used for ocular surface treatments, including DED. Amniotic membrane extract ophthalmic solution contains growth factors more similar to the composition of native human tears than the aforementioned forms and has anti-inflammatory and immunomodulatory properties [115]. It also has the advantage of being immunologically inactive due to the absence of human leukocyte antigens A, B, C, and DR, while expressing CD59, a negative regulator of complement activation, and human leukocyte antigen G. Amniotic membrane extract ophthalmic solution has been reported to be useful in severe DED [115,116]. Foreign body and ocular burning sensations have been reported as side-effects of this treatment [117].

### 5.7. Lutein Supplement

Lutein has become widely used in recent years as a dietary supplement for the prevention and treatment of age-related macular degeneration, a common cause of central vision loss in the elderly. Lutein is a macular pigment carotenoid that selectively binds to the macula and has antioxidant properties due to the conjugated double bonds and hydroxyl groups in the polyene chain [118]. Recently, an anti-inflammatory effect by downregulating nuclear factor-kappa B p65 activity, thereby decreasing cyclooxygenase-2 and nitric oxide synthase expression, was also reported [119]. In addition to this, improvement of visual function, contrast sensitivity, and physiological function modulation effects have also been observed [120]. Based on the above facts, it may be effective in the treatment of DED, but clinical results have not yet been shown. Future reports are expected.

### 5.8. Laser Acupuncture

Acupuncture is proven to be beneficial when treating DED [121]. Acupuncture regulates the autoregulatory and immunological systems through expanding vascularity and augmenting neuropeptides [122]. During DED therapy, it ameliorates tear film instability via enhanced tear protein production, modulation of hormone concentrations and lacrimal gland metabolites, enhancement of the content of acetylcholine in the lacrimal gland, modulation of angioactive enteropeptides, and a decrease in pro-inflammatory cytokines in the ocular surface [123,124]. Laser acupuncture is a fusion of Chinese medicine’s pathway and acupoint theory with modern laser therapy techniques. Instead of metallic needles, this technology uses nonthermal, low-intensity laser radiation to activate acupuncture sites, making the treatment short, painless, sterile, and noninvasive [125]. Complications of conventional acupuncture (fainting, breaking, bending, and stinging) can likewise be averted. Accordingly, it has been indicated that laser acupuncture is a helpful supplemental treatment from the standpoint of efficacy and safety in cases where eyedrops treatment is ineffective [123]. Yet, laser acupuncture therapy remains in need of a lot of optimization and adjustment regarding therapeutic parameters and the design of the laser equipment, and laser acupuncture mechanisms also require more exploration. Skin irritation, albeit minor, also occurs as a side-effect.

## 6. Discussion and Conclusions

Each report shows the new generation DED eye drops are superior to artificial tears and sodium hyaluronate, making mild DED controlled. For the short tear film breakup time-type DED, the goal of treatment is to stabilize the tear film, so the first option is to administer mucin-secreting eye drops. Diquafosol, an eye drop that stimulates mucin secretion, increases mucin, water, and lipids, and is expected to be effective in treating not only DED with short tear film breakup time-type but also aqueous-deficient-type DED. The long-acting diquafosol, which has recently become available, has a recommended frequency of eye drops of three times a day and is expected to be effective in terms of adherence. In addition to this, at the start of DED treatment, I suggest oral administration of supplements in combination with the eye drops. The supplement is applicable to all types of DED as well as diquafosol because of its moisturizing and anti-inflammatory effects, as well as its potential to improve eye strain. And if the disease is difficult to control even with this combination therapy, local anti-inflammatory treatment is necessary. In such conditions, the restoration of tear film homeostasis is inadequate, and the ocular surface continues to be exposed to persistent oxidative stress and other factors. Macrophages and monocytes are activated to promote epithelial cell recovery, and autophagy to maintain ocular surface homeostasis is decreased [126]. This indicates a state of ongoing inflammation of the ocular surface and its damage has not recovered [127,128]. Therefore, additional treatment is suggested with cyclosporine eye drops. With these combination treatments, the osmotic pressure increases in the tear film and much of the ocular surface inflammation can be controlled. Although there is a problem of adherence to additional medications at this time, cyclosporine eye drops are relatively easy to introduce because they require fewer drops than diquafosol. However, there are cases of DED that are difficult to control even with the previous treatments, in which case additional treatments (adjunctive therapies) for the causative disease should be considered. I suggest the use of therapeutic contact lenses in cases of severe epithelial damage, additional treatment with human umbilical cord serum eye drops for post-ocular surgery DED and severe DED, and additional treatment with intense pulsed light, lid debridement, vectored thermal pulsation system or intraductal meibomian gland probing for meibomian gland dysfunction.

The new generation eye drops presented in this review are not approved in all countries. In addition, many facilities may not have adjunctive therapies in place. Therefore, I will supplement those alternative therapies. As an alternative to mucin secretagogues, artificial tears and/or the combination of sodium hyaluronate ophthalmic solution are suggested. Particularly with regard to artificial tears, artificial tears containing viscosity-enhancing agents or oily agents and surfactants are effective in stabilizing the tear film [20]. However, alternative eye drops require frequent instillation, so special attention must be paid to adherence and continuity of treatment. Steroid eye drops are recommended as anti-inflammatory eye drops with caution regarding side-effects, and as an alternative adjunctive therapy. Bandage contact lenses and/or autologous serum ophthalmic solutions may be effective for severe ocular surface disorders, and eyelid hygiene with warm compresses and eyelid massage may be useful for meibomian gland dysfunction. Frequent blinking, protective eyewear, and environmental modifications (e.g., use of humidifiers) are also recommended in conjunction with these treatments, as is also true for the new generation therapies.

As DED is a chronic disease, a key challenge for future DED therapy is to achieve better therapeutic persistence. A report states that only approximately 10% of DED sufferers are using therapeutic eye drops as per the number of times recommended in the accompanying document [36]. Additional research is required to ensure favorable adherence. In addition, approval of the therapies presented in the Expected future therapy section in different countries is expected for the advancement of DED treatment.

## 7. Limitations

First, there are few treatments in this review that have published research results supporting their enhancement of the physical properties of human tear fluid. The same is true for diquafosol, which is identified as the first option for DED treatment in this paper. Second, although the treatment protocols for DED proposed in this review appear to be effective, they have not been validated by clinical trials. Further research and controlled clinical trials are needed to address these limitations.

## Figures and Tables

**Table 1:** Major therapy (new generation eye drops).

Name	Principle	Dosage	Application Frequency	Therapeutic Effect	Limitation
Diquafosol	Mucin and water secretion promotion	Eye drops	3 times a day *	Stabilization of tear film and increased tear fluid	Irritation
Cyclosporine	Inflammation suppression	Eye drops	1 times a day **	Anti-inflammatory and reduction of tissue damage	Heat and stinging pain

* Long-acting diquafosol; ** Ikervis^®^.

**Table 2:** Adjunctive therapy.

Name	Dosage	Therapeutic Effect	Limitation
Supplement	Internal medicine	Moisturising and anti-inflammatory	Intraocular bioavailability
Therapeutic contact lens	Contact lens	Corneal healing promotion and drug delivery system	Infection
Human umbilical cord serum eye drops	Eye drops	Anti-inflammatory	Allergy and infection
Intense pulsed light	Myobomian gland dysfunction therapy	Anti-inflammatory	Skin bruising
Lid debris debridement		Improved mybum characteristics	Irritation, erythema, and abrasion
Vectored thermal pulsation system		Relief of obstruction of the meibomian glands	Skin pain and swelling
Intraductal meibomian gland probing		Relief of obstruction of the meibomian glands	Bleeding

**Table 3:** Expected future therapy.

Name	Dosage	Therapeutic Effect	Limitation
Tacrolimus ophthalmic solution	Eye drops	Anti-inflammatory	Tingling and burning sensations
Tanfanercept ophthalmic solution		Anti-inflammatory	Intraocular bioavailability
Lifitegrast ophthalmic solution		Anti-inflammatory	Taste disturbance
Perfluorohexyloctane ophthalmic solution		Lipid layer thickening and cooling on ocular surface	Hypersensitivity
Lactoferrin ophthalmic solution		Anti-inflammatory	Intraocular bioavailability
Amniotic membrane extract ophthalmic solution		Anti-inflammatory	Foreign body and ocular burning sensations
Lutein supplement	Internal medicine	Anti-inflammatory	Intraocular bioavailability
Laser acupuncture	Acupuncture	Anti-inflammatory	Skin irritation

## Data Availability

Not applicable.
